# Complementary Feeding Social and Behavior Change Communication for Fathers and Mothers Improves Children's Consumption of Fish and Eggs and Minimum Meal Frequency in Kaduna State, Nigeria

**DOI:** 10.1093/cdn/nzac075

**Published:** 2022-04-08

**Authors:** Valerie L Flax, Abiodun Ipadeola, Courtney H Schnefke, Sarah Kwasu, Abdulrahaman A Mikail, Sujata Bose, Alice O Brower, Susan Edwards

**Affiliations:** RTI International, Research Triangle Park, NC, USA; Datametrics Associates Ltd., Abuja, Nigeria; RTI International, Research Triangle Park, NC, USA; Alive & Thrive Nigeria, Kaduna, Nigeria; I Care Women and Youth Initiative (ICARE), Kaduna, Nigeria; Alive & Thrive, Washington, DC, USA; RTI International, Research Triangle Park, NC, USA; RTI International, Research Triangle Park, NC, USA

**Keywords:** complementary feeding, dietary diversity, meal frequency, children, fathers, mothers, social behavior change communication, mHealth, Nigeria, sub-Saharan Africa

## Abstract

**Background:**

Fathers are key influencers of complementary feeding practices, but few studies in low- and middle-income countries have measured the effects of complementary feeding social and behavior change communication (SBCC) targeted at both fathers and mothers.

**Objectives:**

The aims of this study were to measure the effects of an SBCC intervention on children's dietary diversity (primary outcome) and other complementary feeding indicators, fathers’ and mothers’ complementary feeding knowledge, and fathers’ support for complementary feeding (secondary outcomes).

**Methods:**

The 12-mo intervention in Kaduna State, Nigeria, engaged parents through community meetings, religious services, home visits from community health extension workers (CHEWs), mobile phone messages (fathers only), and mass media. Cross-sectional population-based surveys of cohabiting fathers and mothers with a child aged 6–23 mo were conducted, and regression models were used to compare results at baseline (*n* = 497) and endline (*n* = 495).

**Results:**

Children's minimum dietary diversity did not change from baseline to endline (62% to 65%, *P *= 0.441). Children's consumption of fish (36% to 44%, *P *= 0.012) and eggs (8% to 20%, *P *= 0.004) and minimum meal frequency (58% to 73%, *P *< 0.001) increased. Fathers’ and mothers’ knowledge of the timing of introduction of different foods and meal frequency improved. Fathers’ support for child feeding by providing money for food increased (79% to 90%, *P *< 0.001). Fathers’ and mothers’ reported intervention exposure was low (11–26% across types of SBCC). Child feeding outcomes were not associated with fathers’ exposure. Children's odds of both fish and egg consumption increased significantly with mothers’ exposure to community meetings, religious services, home visits, and television spots, and children's odds of minimum meal frequency increased significantly with mothers’ exposure to home visits.

**Conclusions:**

A multipronged SBCC intervention improved complementary feeding practices, fathers’ and mothers’ knowledge of complementary feeding, and fathers’ support for complementary feeding, despite low levels of reported exposure, which may have been influenced by coronavirus disease 2019 (COVID-19) disruptions. This trial was registered at ClinicalTrials.gov as NCT04835662.

## Teaser

A multicomponent social and behavior change communication intervention for fathers and mothers in northern Nigeria increased children’s consumption of fish and eggs and minimum meal frequency.

## Introduction

At the age of 6 mo, breast milk alone is no longer sufficient to meet an infant's nutrient needs; consequently, foods and liquids to complement the nutrients from breast milk are required to maintain child growth and development (1, 2). The WHO recommends that children aged 6 to 23 mo eat a minimum number food groups per day (minimum dietary diversity) to ensure adequate dietary quality and consume a sufficient number of meals per day depending on their age and breastfeeding status (minimum meal frequency) to ensure adequate macronutrient intake (3, 4). However, many children globally are not fed according to these guidelines. For example, among children aged 6 to 23 mo in West and Central Africa, only 19% achieved minimum dietary diversity and 47% achieved minimum meal frequency (5).

Multiple factors affect complementary feeding practices, including parental knowledge, attitudes, and beliefs related to feeding practices; funds available for complementary foods; household decision making about food purchases; influence of family members; time available for food preparation and child feeding; and demographic factors (6). Mothers are often the target group for social and behavior change communication (SBCC) interventions intended to improve complementary feeding practices and the factors that contribute to them because they are the main caregivers of young children and are usually responsible for purchasing and preparing food. Previous studies that provided mothers in low- and middle-income countries (LMICs) with nutrition education or SBCC related to complementary feeding showed that such interventions can increase maternal knowledge and improve child feeding practices, with increases in dietary diversity, consumption of specific foods, and meal frequency, ranging from approximately 5 to more than 50 percentage points (6–14). However, other family members, especially fathers and grandmothers, are key influencers of child feeding practices (15–19). They can play important roles in complementary feeding by providing informational, emotional, and instrumental social support for feeding to the mother or by performing specific complementary feeding tasks, such as purchasing or preparing food or directly feeding the child (20). They also can make it more challenging for mothers to follow recommended complementary feeding practices if they are unsupportive (21). Involving fathers in complementary feeding interventions could help increase intervention impact, especially in contexts where there are distinct gender roles and men have control over household finances and decision making. To our knowledge, only 1 published study, conducted in Kenya, has shown quantitatively that a nutrition SBCC intervention that engaged fathers had an impact on complementary feeding practices (22).

The Alive & Thrive initiative in Nigeria is working to scale up infant and young child feeding (IYCF) interventions and increase optimal feeding practices through interpersonal communication from health workers, community mobilization activities conducted by religious and community leaders, and mass media campaigns. A baseline population-based survey conducted in Kaduna State in 2017 for Alive & Thrive's overall program evaluation showed that 22% of children aged 6 to 23 mo achieved minimum dietary diversity and 76% achieved minimum meal frequency (23). Qualitative formative data, also collected in 2017, indicated that fathers in Kaduna State are key influencers of IYCF practices and play an important role in providing money for or directly purchasing food for the child (24). However, fathers are not well informed about the types of foods that children need to grow and develop or the necessity of providing enough resources to procure those foods (23).

Alive & Thrive sought to address these gaps by designing an intervention to engage fathers in Kaduna State to support child dietary diversity and other complementary feeding practices. The main aim of the study was to measure the effect of the intervention on children's dietary diversity, which was the primary outcome. The secondary aim was to measure changes in other complementary feeding indicators and in factors on the pathway between the intervention and the outcomes, including fathers’ and mothers’ complementary feeding knowledge and fathers’ support for complementary feeding.

## Methods

### Study setting

The study was conducted in 6 of 12 wards in the Igabi local government area (LGA) of Kaduna State. An LGA is the largest administrative subunit of a state in Nigeria; LGAs are further subdivided into administrative wards. Igabi LGA was chosen because it includes urban and rural areas and a mix of religious affiliations (Muslim and Christian) and ethnic groups (Hausa, Fulani, and others). Agriculture is the main source of livelihood in the LGA. Food availability varies by season, with agricultural products more plentiful toward the end of the rainy season and beginning of the dry season.

### Study design

This study was a pre-post evaluation of Alive & Thrive's intervention in the Igabi LGA to improve dietary diversity and other complementary feeding indicators among children aged 6 to 23 mo by encouraging fathers to support complementary feeding among their young children. We conducted cross-sectional surveys of fathers and mothers before and after the 12-mo intervention.

### Intervention

**FIGURE 1:**
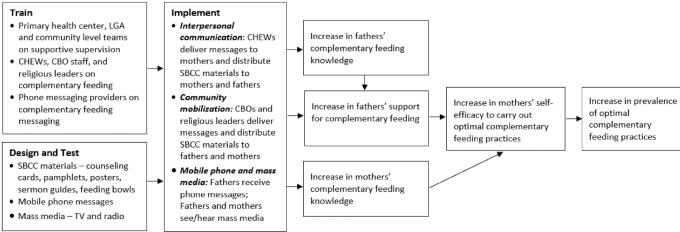
Theory of change for the Igabi LGA Alive & Thrive complementary feeding study. CBO, community-based organization; CHEW, community health extension worker; LGA, local government area; SBCC, social and behavior change communication; TV, television.

**Figure 1** shows the theory of change for the intervention, which is based on the SBCC strategy tested by Alive & Thrive in several countries (25). The theory of change posits that interpersonal communication, community mobilization by religious and community leaders, and mobile phone and mass media messages will increase fathers’ and mothers’ complementary feeding knowledge and increase fathers’ support for complementary feeding. Increases in fathers’ support for and mothers’ knowledge of complementary feeding will improve mothers’ self-efficacy to carry out optimal complementary feeding practices, leading to increased prevalence of the optimal practices. The intervention was designed based on formative research. Its goal was to mobilize and motivate fathers of children aged 6 to 23 mo to support optimal complementary feeding practices by providing money for or procuring specific nutritious foods for complementary feeding and encouraging mothers to follow the WHO minimum dietary diversity and minimum meal frequency guidelines (3). Although the intervention targeted fathers, it was also designed to reach mothers, as the primary caregivers of young children. Several nutritious foods were specifically promoted as part of the intervention, including eggs, fish, beans, pumpkin, sweet potatoes, and spinach. The intervention used a variety of SBCC materials, including sermon guides, counseling cards, pamphlets, posters, and feeding bowls (**Supplemental Materials 1**). Television (TV) and radio spots that were part of Alive & Thrive's overall intervention were also available to participants in this study.

Fathers who owned mobile phones received weekly mobile phone messages on complementary feeding through short message service (SMS) text messages and voice prompts (prerecorded voice messages) promoting dietary diversity (**Supplemental Table 1**). Fathers were also reached with messages on complementary feeding through religious leaders and community-based organizations (CBOs). Religious leaders (14 imams and 4 pastors) were trained on complementary feeding and given sermon guides they could use during religious services or religious gatherings—such as naming ceremonies and weddings—where fathers were present. Additional messaging from staff of 13 CBOs was intended to complement the other activities targeting fathers. The leaders of the CBOs were trained on complementary feeding SBCC, including the use of the pamphlets and counseling cards. During their regular CBO meetings, they included short discussions about complementary feeding before starting other activities and provided the pamphlets to members. Complementary feeding posters were placed in strategic locations in communities, including at CBO meeting points, in places of worship, and in health facilities. Although the complementary feeding information shared during religious services and CBO meetings was targeted at fathers, mothers could also participate and hear the messages through these channels.

A total of 60 community health extension workers (CHEWs) were trained to conduct at least 2 home visits with each mother of a child aged 6 to 23 mo and were given complementary feeding counseling cards to serve as a guide during their visits. They delivered age-specific complementary feeding messages, provided each household with the Alive & Thrive feeding bowl (which shows diverse nutritious foods and has markings indicating the quantity of food to feed the child at different ages), and gave copies of the pamphlets to mothers, fathers, and other family members present. If fathers were at home, CHEWs included them in the discussions of complementary feeding during their visits.

The I Care Women and Youth Initiative (ICARE) was responsible for intervention implementation. ICARE staff canvassed the wards and created lists of fathers and their phone numbers. Fathers were added or removed from the phone messaging list as their child entered or left the target age range (6–23 mo). ICARE provided supportive supervision to CHEWs, CBOs, and religious leaders involved in the intervention. During the intervention period, ICARE compiled monthly tallies of the number of mothers and fathers counseled during home visits, number of fathers counseled during CBO meetings, number of people attending sermons, and number of fathers who received phone messages.

### Sampling and participant eligibility

Six administrative wards in Igabi LGA were purposefully selected to represent urban (Rigasa and Rigachikun wards) and rural areas (Igabi, Turunku, Kwarau, and Zangon Aya wards) and to consider variations in ethnic groups, religion, and presence of CBOs. A list of communities with their population size was obtained from the Kaduna State Primary Health Development Agency and served as the basis for multistage cluster sampling. Communities within the sampling frame were divided into strata by ward and urban/rural location. At baseline, communities were randomly sampled proportional to the population size of the strata. At endline, a new sample of communities was drawn following the same procedures. Randomly selected replacement communities of similar population size within the same stratum were used at baseline when some selected communities did not have enough eligible households and were also used at both baseline and endline when flooding limited accessibility to some communities. Within communities selected for the surveys, enumerators were trained to conduct random route walks to identify and enroll 5 households.

Men were eligible to participate in the surveys if they were aged 18 y or older, had a child aged 6 to 23 mo, and were living in the same household as the child's mother. Women were eligible to participate if they were aged 18 to 49 y or aged 15 to 17 y and married, had a child aged 6 to 23 mo, and were living in the same household as the child's father. Under Nigerian law, women are considered adults and can consent for themselves if they are aged 15 y or older and married. Women are adults regardless of marital status when they are aged 18 y or older. Fathers and mothers were enrolled as pairs. If there was more than 1 eligible woman in the household, one of them was randomly selected. Signed informed consent was obtained from participants. Each father–mother pair was given an incentive of 1200 Naira after completing their interviews. The study was approved by the Kaduna State Health Research Ethics Committee and the RTI International Institutional Review Board and registered with ClinicalTrials.gov (NCT04835662).

### Measures

All complementary feeding indicators were measured as part of the mother's questionnaire using the WHO IYCF questionnaire (26). These data were collected only from mothers because they are responsible for child feeding in this context. Minimum dietary diversity was the main study outcome. The 2021 WHO and UNICEF guidelines for calculating minimum dietary diversity (including breast milk as a food group) were not available when the study was designed and conducted (4). Therefore, minimum dietary diversity was defined using the WHO 2008 guidelines, as children consuming ≥4 out of 7 food groups (grains, roots, and tubers; legumes and nuts; dairy; flesh foods; eggs; vitamin A–rich fruits and vegetables; and other fruits and vegetables) on the previous day (3).

Several other complementary feeding practices were measured as secondary outcomes using the WHO IYCF indicators, including specific food groups, minimum meal frequency, and minimum acceptable diet. The 7 food groups that make up minimum dietary diversity were examined individually, with flesh foods calculated both overall (including meat, poultry, and fish) and with fish as a separate category, because fish was specifically promoted by the intervention. Minimum meal frequency was defined as children receiving solid, semi-solid, or soft foods at least 2 times on the previous day if they were aged 6–8 mo and breastfed, at least 3 times if they were aged 6–23 mo and breastfed, or at least 4 times if they were aged 6–23 mo and not breastfed. Minimum acceptable diet was defined as children who have achieved both minimum dietary diversity and minimum meal frequency. Age in months when the child started eating specific foods also was reported by mothers. Other secondary outcomes included fathers’ and mothers’ complementary feeding knowledge, fathers’ support for complementary feeding, and mothers’ perceptions of fathers’ support. Knowledge and support questions were measured without providing response options.

Fathers’ and mothers’ exposure to the intervention was based on self-report. Intervention exposures included attending community meetings or religious services where feeding children a variety of foods was discussed, participating in home visits from a CHEW, seeing child feeding information on TV or hearing it on the radio, and receiving text/SMS messages or voice prompts about child feeding (fathers only). The reference period for community and home exposures was the past 6 mo; the reference period for mass media and phone exposures was the past 30 d.

Sociodemographic characteristics of the households and participants were collected using questions from the Nigeria Demographic and Health Survey (27). An asset score was created based on ownership of 39 items, including durable goods and livestock. Household food insecurity was measured using the Household Hunger Scale (28).

### Sample size

We calculated the sample size for the cross-sectional surveys using our main outcome variable, minimum dietary diversity. To detect an 8-percentage-point change (from 22% to 30%) in minimum dietary diversity from baseline to endline with 80% power, α = 0.05, intraclass correlation coefficient (ICC) = 0.2, and 5 households per community or cluster, 495 men and 495 women were required in each survey. An 8-percentage-point increase was selected by Alive & Thrive based on experience implementing similar types of programs in other countries (12, 13).

### Data collection

The baseline survey was conducted in June 2019 and the endline survey was conducted from August to September 2020. Experienced interviewers were trained on data collection procedures before each survey. Both trainings included a pilot of data-collection procedures. The data-collection team was divided into groups with 2 male and 2 female interviewers per supervisor. Interviewers were paired by gender so that male interviewers interviewed fathers and female interviewers interviewed mothers. Interviews with the father and mother in the same household were conducted separately. Data were entered into digital tablets using the the ODK Collect open-source data collection app. Data were anonymized after linking the fathers’ and mothers’ data for analysis. Security of the identifiable data was the responsibility of Datametrics Associates and RTI International.

### Data analysis

The analysis was conducted in Stata MP Version 16.0 (StataCorp) using survey commands to account for the survey design. At each round of data collection, the data were analyzed cross-sectionally to determine population-level estimates of minimum dietary diversity, consumption of specific food groups, minimum meal frequency, minimum acceptable diet, and knowledge of and support for complementary feeding. Differences in population estimates between baseline and endline were evaluated using logistic regression for binary variables and linear regression for continuous variables. Overall tests of differences for multinomial variables were conducted using either multinomial logistic regression or ordinal logistic regression, depending on the structure of the multinomial variable. *P* values are presented based on the overall Wald test for the impact of data-collection wave on the modeled variable. For overall models, levels with less than 5 respondents at 1 time point were collapsed with other categories.

At baseline and endline, the sample was weighted to be representative of households with a father and at least 1 mother with a child aged 6 to 23 mo in Igabi LGA. Base weights for the household, fathers in the household, and mothers in the household were adjusted for nonresponse. Inverse probability weights (IPWs) were used to adjust for unintentional bias because of the lack of a comparison group and random assignment. Adjustments were made based on differences in population-level estimates of demographic characteristics between the baseline and endline samples. Parent and household characteristics with differences at the *P *< 0.10 level for either fathers or mothers were included in the initial propensity score models. Using backward selection at a *P *< 0.05 level, the covariate with the largest *P* value was removed one-by-one until all variables in the final model were statistically significant. The final propensity models for fathers and mothers included the same set of household characteristics: age of the head of the household; household composition counts for adults, children aged less than 5 y, and school-aged children; sharing a toilet; main source of household's drinking water; type of toilet facility; and urbanicity. Additional covariates included educational level for fathers and ethnicity for mothers. For any respondent missing at least 1 of the controlled-for covariates, no IPW adjustment was made to the original survey weights; this accounted for approximately 4% of each sample. The final adjustment was implemented by multiplying the IPW estimate by the base survey weight. These adjusted weights were used to estimate adjusted *P* values throughout the paper.

## Results

**FIGURE 2:**
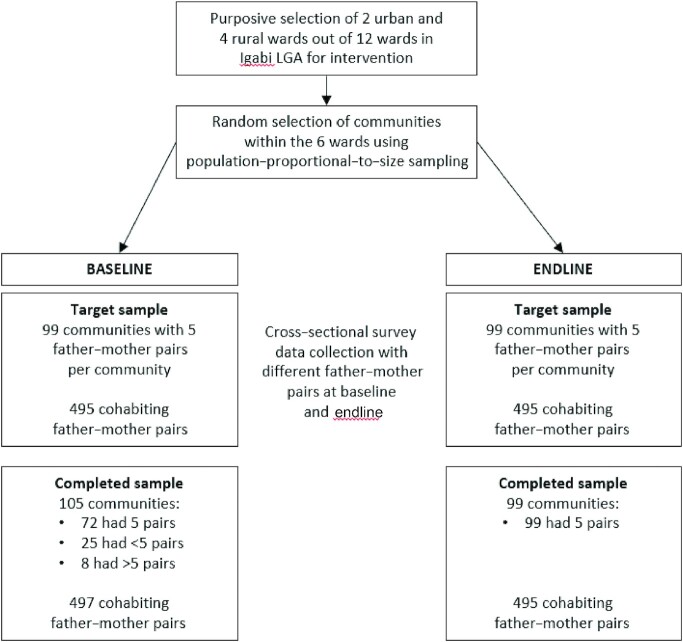
Study flow diagram for the Igabi LGA Alive & Thrive complementary feeding study. LGA, local government area.

The flow of communities and participants in the study is shown in **Figure 2**. At baseline, extra communities were included because some communities did not have 5 eligible households, and the final baseline sample was 497 father–mother pairs. At endline, the target number of communities and participants per community was achieved, resulting in a final endline sample of 495 father–mother pairs.

### Household and participant characteristics

Household and participant characteristics are shown in **Table 1**. Several characteristics differed between baseline and endline. Compared with baseline, households at endline had a slightly smaller household size, slightly fewer adults aged 18 y or older, fewer school-aged children, more children aged less than 5 y, fewer rooms in their homes, and a higher asset score. The mean age for fathers was higher at endline compared with baseline, whereas the mean age for mothers and children did not differ at endline compared with baseline. Sources of drinking water and type of toilet facility differed between baseline and endline. Fewer households shared a toilet facility at endline compared with baseline. More households had moderate household hunger at endline compared with baseline, although most households at both time points had little to no household hunger. Fewer fathers and mothers were from the Fulani ethnic group at endline compared with baseline. Fathers’ occupation differed between baseline and endline, with fewer fathers having salaried government jobs and more working as small traders or in self-employment at endline. More fathers and mothers at endline compared with baseline had completed some postsecondary education.

**TABLE 1:** Sociodemographic characteristics of households and participants in the Igabi LGA Alive & Thrive complementary feeding study^1^

	Baseline (*n* = 497)		Endline (*n* = 495)		Unadjusted *P* value
	Mean	SE	Mean	SE	
Household size, *n*	5.5	0.1	5.2	0.1	0.066
Number of adults aged ≥18 y	2.5	0.1	2.4	0.0	0.043
Number of school-aged children^2^	2.0	0.1	1.7	0.1	0.016
Number of children aged <5 y	1.3	0.0	1.6	0.0	<0.001
Number of rooms in house	3.5	0.1	3.2	0.1	0.032
Asset score^3^	9.9	0.2	10.4	0.2	0.090
Father's age, y	35.3	0.4	36.6	0.4	0.033
Mother's age, y	25.4	0.3	25.7	0.3	0.432
Child's age, mo	14.2	0.3	14.0	0.3	0.588
	**%**	***n***	**%**	***n***	
Urban (vs. rural)	62	280	60	283	0.027
Polygamous household	17	91	21	99	0.136
Main source of household's drinking water					<0.001
Piped into dwelling or yard/plot	6	28	21	100	
Public tab/standpipe	13	59	12	60	
Tube well or borehole	38	191	30	152	
Protected dug well	33	165	25	123	
Unprotected dug well	7	43	10	51	
Other	3	11	2	9	
Type of toilet facility					<0.001
Flush or pour toilet	28	134	45	225	
Pit latrine with slab	54	265	32	156	
Pit latrine without slab/open pit	15	83	20	102	
Other	3	14	2	12	
Toilet facility is shared with other households	53	253	33	166	<0.001
Household hunger scale					0.028
Little to no household hunger	94	467	90	447	
Moderate household hunger	5	27	9	45	
Severe household hunger	1	3	1	3	
Father's ethnicity					0.068
Hausa	87	435	88	437	
Fulani	10	52	7	36	
Other	3	10	5	22	
Father's occupation					0.044
Farmer	28	157	28	140	
Salaried government employee	8	42	12	55	
Salaried nongovernment employee	9	42	8	40	
Small trader/self-employment	53	241	47	229	
Other	1	7	3	16	
Father's education					<0.001
Never attended school	17	91	13	61	
Primary	27	128	17	80	
Secondary	40	187	42	204	
Postsecondary	13	67	21	104	
Vocational	3	15	0	1	
Mother's ethnicity					0.001
Hausa	82	409	84	418	
Fulani	13	71	7	36	
Other	5	17	9	41	
Mother's occupation					0.713
Salaried government employee	1	5	1	5	
Salaried nongovernment employee	2	11	1	7	
Small trader/self-employed	50	235	53	259	
Unemployed	47	241	43	217	
Mother's education					0.016
Never attended school	30	148	25	122	
Primary	33	166	33	164	
Secondary	33	161	33	166	
Postsecondary	2	10	6	32	

1 LGA, local government area.

2 School-aged children = children aged 3 to 16 y.

3 Average number of assets participant households owned out of 39 items listed on the survey questionnaire.

### Complementary feeding practices

Children's mean dietary diversity scores were the same at baseline and endline (3.9 out of 7 food groups). The percentage of children with minimum dietary diversity was slightly higher at endline, but the difference was not statistically significant (62% to 65%, *P *= 0.358) (**Table 2**). However, children's consumption of fish (36% to 44%, *P *= 0.012) and eggs (8% to 20%, *P *= 0.004) was significantly higher at endline compared with baseline. A higher percentage of children at endline compared with baseline had minimum meal frequency (58% to 73%, *P *< 0.001) and minimum acceptable diet (40% to 51%, *P *< 0.001). The mean age in months of introduction of several foods was lower at endline compared with baseline, putting it closer to the recommended timing of introduction at 6 mo for tubers (8.4 to 7.6 mo, *P *= 0.014), beans (7.3 to 6.8 mo, *P *= 0.019), green leafy vegetables (8.1 to 7.2 mo, *P *= 0.002), vitamin A–rich fruits and vegetables (9.2 to 7.9 mo, *P *< 0.001), meat or poultry (9.6 to 8.3 mo, *P *< 0.001), fish (7.6 to 6.9 mo, *P *= 0.003), eggs (7.3 to 6.6 mo, *P *= 0.012), and nuts (9.5 to 8.1 mo, *P *= 0.001).

**TABLE 2:** Complementary feeding practices before and after the intervention in the Igabi LGA Alive & Thrive complementary feeding study^1^

	Baseline (*n* = 497)		Endline (*n* = 495)		*P* value	
	%	*n*	%	*n*	Unadjusted	Adjusted^2^
Minimum dietary diversity	62	313	65	315	0.347	0.441
Food-group consumption (24 h)						
Grains, roots, and tubers	100	497	92	451	—	—
Legumes and nuts	70	346	68	334	0.719	0.994
Dairy products	44	219	47	233	0.408	0.907
Flesh foods	44	218	50	248	0.069	0.069
Fish (subset of flesh foods)	36	180	43	214	0.021	0.016
Eggs	8	46	20	96	<0.001	0.003
Vitamin A–rich fruits and vegetables	36	185	31	151	0.143	0.164
Other fruits and vegetables	90	444	80	395	<0.001	0.001
Minimum meal frequency	58	290	73	357	<0.001	<0.001
Minimum acceptable diet	40	209	51	248	0.001	<0.001
	**Mean**	**SE**	**Mean**	**SE**		
Reported timing of introduction of foods, mo						
Cereals	6.5	0.1	6.4	0.1	0.509	0.285
Tubers	8.4	0.2	7.6	0.1	<0.001	0.014
Beans	7.3	0.2	6.8	0.1	0.003	0.019
Green leafy vegetables	8.1	0.2	7.2	0.1	<0.001	0.002
Vitamin A–rich fruits and vegetables	9.2	0.2	7.9	0.1	<0.001	<0.001
Other fruits and vegetables	7.8	0.2	7.5	0.1	0.133	0.242
Meat or poultry	9.6	0.2	8.3	0.1	<0.001	<0.001
Fish	7.6	0.2	6.9	0.1	<0.001	0.003
Eggs	7.3	0.2	6.6	0.1	0.001	0.012
Nuts	9.5	0.2	8.1	0.1	<0.001	0.001
Dairy products	6.7	0.2	6.4	0.1	0.063	0.094

1 LGA, local government area.

2 Adjusted for age of head of household; household composition counts for adults, children aged <5 y, and school-aged children; sharing a toilet; main source of household's drinking water; type of toilet facility; urbanicity; and mothers' ethnicity using an inverse probability weighting.

### Complementary feeding knowledge

Several elements of complementary feeding knowledge differed significantly between the baseline and endline for both fathers and mothers (**Table 3**). In general, mothers’ knowledge of the timing of introduction of specific foods was closer to the recommended age of 6 mo than fathers for all types of food. The mean age at which fathers and mothers said specific foods should be introduced to children was significantly lower at endline compared with baseline, moving closer to the recommended age of 6 mo for cereals (fathers, mothers), tubers (fathers, mothers), beans (fathers, mothers), green leafy vegetables (fathers, mothers), vitamin A–rich fruits and vegetables (fathers, mothers), other fruits and vegetables (fathers), meat or poultry (fathers, mothers), fish (fathers, mothers), eggs (fathers, mothers), nuts (mothers), and dairy products (mothers). Fathers’ knowledge of the mean number of times per day a breastfed child aged 9 to 23 mo should eat decreased slightly from baseline to endline. The percentage of fathers who named specific food groups as contributing to children aged 6 to 23 mo eating a variety of foods was lower at endline as compared with baseline for dairy products but not did not differ from baseline to endline for other types of food. The percentage of mothers who named specific food groups as contributing to children aged 6 to 23 mo eating a variety of foods was lower at endline as compared with baseline for cereals and beans, and higher for tubers, green leafy vegetables, and meat or poultry.

**TABLE 3:** Fathers’ and mothers’ knowledge of complementary feeding before and after the intervention in the Igabi LGA Alive & Thrive complementary feeding study^1^

	Baseline (*n* = 497)		Endline (*n* = 495)		*P* value	
	Mean	SE	Mean	SE	Unadjusted	Adjusted^2^
Father						
Age of introduction of foods, mo						
Cereals	8.6	0.2	7.3	0.1	<0.001	0.001
Tubers	11.8	0.3	9.5	0.2	<0.001	<0.001
Beans	10.9	0.2	8.7	0.2	<0.001	<0.001
Green leafy vegetables	10.2	0.2	9.0	0.2	<0.001	0.002
Vitamin A–rich fruits and vegetables	11.1	0.2	9.7	0.2	<0.001	<0.001
Other fruits and vegetables	10.3	0.2	9.5	0.2	0.009	0.031
Meat or poultry	12.7	0.3	10.5	0.2	<0.001	<0.001
Fish	9.9	0.2	8.5	0.1	<0.001	<0.001
Eggs	8.6	0.2	8.0	0.1	0.007	0.011
Nuts	12.6	0.3	11.7	0.3	0.047	0.195
Dairy products	6.6	0.2	7.0	0.1	0.162	0.104
Times per day breastfed child 6 to 8 mo should eat	3.7	0.1	3.4	0.3	0.441	0.827
Times per day breastfed child 9 to 23 mo should eat	3.8	0.1	3.5	0.1	<0.001	<0.001
	**%**	***n***	**%**	***n***		
Food groups important to help children 6 to 23 mo old eat a variety of foods						
Cereals	70	337	75	374	0.119	0.114
Tubers	38	192	46	232	0.010	0.318
Beans	54	265	61	304	0.024	0.781
Green leafy vegetables	39	199	46	225	0.040	0.440
Vitamin A–rich fruits and vegetables	39	202	36	176	0.327	0.293
Other fruits and vegetables	41	201	36	176	0.176	0.053
Meat or poultry	28	150	28	138	0.947	0.403
Fish	57	281	58	282	0.740	0.209
Eggs	56	273	51	251	0.122	0.627
Nuts	26	140	31	156	0.056	0.327
Dairy products	48	138	42	206	0.068	0.023
	**Mean**	**SE**	**Mean**	**SE**		
Mother						
Age of introduction of foods, mo						
Cereals	7.0	0.1	6.5	0.1	<0.001	0.009
Tubers	9.2	0.2	7.7	0.1	<0.001	<0.001
Beans	7.5	0.1	6.9	0.1	<0.001	0.001
Green leafy vegetables	8.6	0.1	7.5	0.1	<0.001	<0.001
Vitamin A–rich fruits and vegetables	9.4	0.2	8.6	0.1	<0.001	<0.001
Other fruits and vegetables	7.8	0.1	7.7	0.1	0.830	0.658
Meat or poultry	10.4	0.2	8.8	0.1	<0.001	<0.001
Fish	8.0	0.2	7.1	0.1	<0.001	0.002
Eggs	7.4	0.1	6.9	0.1	<0.001	0.026
Nuts	10.6	0.2	8.8	0.1	<0.001	<0.001
Dairy products	6.9	0.1	6.5	0.1	0.043	0.045
Times per day breastfed child 6 to 8 months should eat	3.0	0.1	3.2	0.2	0.559	0.882
Times per day breastfed child 9 to 23 months should eat	3.7	0.1	3.7	0.2	0.966	0.381
	**%**	***n***	**%**	***n***		
Food groups important to help children 6 to 23 mo old eat a variety of foods						
Cereals	79	383	74	364	0.087	0.007
Tubers	33	175	53	265	<0.001	<0.001
Beans	77	374	59	291	<0.001	<0.001
Green leafy vegetables	35	186	50	246	<0.001	0.014
Vitamin A–rich fruits and vegetables	2	154	36	169	0.003	0.143
Other fruits and vegetables	31	158	39	189	0.011	0.107
Meat or poultry	20	115	37	181	<0.001	<0.001
Fish	63	320	63	322	0.893	0.642
Eggs	57	290	65	322	0.014	0.264
Nuts	17	93	15	73	0.499	0.861
Dairy products	25	128	27	139	0.589	0.774

1 LGA, local government area.

2 For fathers, adjusted for age of head of household; household composition counts for adults, children aged <5 y, and school-aged children; sharing a toilet; main source of household's drinking water; type of toilet facility; urbanicity; and fathers' level of education using an inverse probability weighting. For mothers, adjusted for age of head of household; household composition counts for adults, children aged <5 y, and school-aged children; sharing a toilet; main source of household's drinking water; type of toilet facility; urbanicity; and mothers' ethnicity using an inverse probability weighting.

### Complementary feeding support

The percentage of mothers who named their husband as a source of child feeding information and advice since the child turned 6 mo old increased from baseline to endline (38% to 51%, *P *= 0.001), whereas the percentage who named their mother or mother-in-law as a source of information or advice was approximately 30% at both time points. The percentage of mothers who stated that their husband is the person who influences their child feeding practices the most also increased (49% to 60%, *P *= 0.047). Approximately three-quarters of mothers at baseline and endline said their husband is the person who currently helps with or supports child feeding the most.

At baseline and endline, both fathers and mothers reported that fathers most commonly support child feeding by providing money for food or purchasing specific foods for the child (**Table 4**). More fathers and mothers reported that fathers provided money for the child's food at endline compared with baseline. Fewer mothers reported that fathers purchased food for the child at endline compared with baseline. More mothers at endline compared with baseline reported that fathers give advice or remind the mother about child feeding and monitor how the mother feeds the child.

**TABLE 4:** Fathers’ support for complementary feeding before and after the intervention as reported by fathers and mothers in the Igabi LGA Alive & Thrive complementary feeding study^1^

	Baseline (*n* = 497)		Endline (*n* = 495)		*P* value	
	%	*n*	%	*n*	Unadjusted	Adjusted^2^
Father						
Complementary feeding support offered by child's father						
No support	1	4	1	7	0.707	0.362
Provides money for child's food	79	386	90	439	<0.001	<0.001
Purchases food for child	61	280	53	262	0.014	0.085
Gives advice or reminds mother about child feeding	13	65	13	64	0.925	0.739
Monitors how mother feeds child	14	62	11	52	0.144	0.075
Feeds child directly	11	50	8	39	0.108	0.447
Teaches child how to feed herself or himself	5	25	6	29	0.663	0.846
Washes child's hands before child eats	12	64	14	72	0.430	0.350
Helps mother with other chores so she can feed or prepare food for child	12	60	10	49	0.305	0.112
Mother						
Complementary feeding support offered by child's father						
No support	2	11	1	7	0.391	0.279
Provides money for child's food	74	355	85	418	<0.001	0.003
Purchases food for child	60	290	30	145	<0.001	<0.001
Gives advice or reminds mother about child feeding	5	32	21	105	<0.001	<0.001
Monitors how mother feeds child	1	6	9	43	<0.001	<0.001
Feeds child directly	25	122	24	116	0.591	0.340
Teaches child how to feed herself or himself	6	37	6	29	0.937	0.803
Washes child's hands before child eats	9	55	7	39	0.438	0.201
Helps mother with other chores so she can feed or prepare food for child	7	43	14	69	0.001	0.081

1 LGA, local government area.

2 For fathers, adjusted for age of head of household; household composition counts for adults, children aged <5 y, and school-aged children; sharing a toilet; main source of household's drinking water; type of toilet facility; urbanicity; and fathers' level of education using an inverse probability weighting. For mothers, adjusted for age of head of household; household composition counts for adults, children aged <5 y, and school-aged children; sharing a toilet; main source of household's drinking water; type of toilet facility; urbanicity; and mothers' ethnicity using an inverse probability weighting.

### Exposure to the intervention and association with child feeding outcomes

Fathers’ self-reported exposure ranged from 11% to 26% and mothers’ self-reported exposure ranged from 12% to 21% for different intervention components (**Table 5**). Among fathers who reported participating in a community meeting or a religious service where complementary feeding was discussed, the topics most of them independently recalled were “introduce complementary foods at 6 months” (community meeting, 66%; religious service, 54%) and “feed the child a variety of foods” (community meeting, 65%; religious service, 52%) (data not shown). Among mothers who reported participating in a home visit from a CHEW, a community meeting, or a religious service, the messages they commonly recalled were “feed the child eggs or fish with mashed sweet potato or pumpkin and mashed beans or spinach” (CHEW visit, 47%; community meeting, 60%; religious service, 59%), “fish and eggs help with the child's brain development” (CHEW visit, 47%; community meeting, 58%; religious service, 41%), and “pumpkin, yellow sweet potato, and spinach protect the child from illness” (CHEW visit, 35%; community meeting, 41%; religious service, 45%) (data not shown).

**TABLE 5:** Fathers’ and mothers’ reported exposure to the complementary feeding intervention at endline in the Igabi LGA Alive & Thrive complementary feeding study^1^

			Association of intervention exposure with child feeding outcomes^2^									
	Intervention exposure (*n* = 495)		Minimum dietary diversity		Consumption of fish		Consumption of eggs		Minimum meal frequency		Minimum acceptable diet	
	%	*n*	aOR	95% CI	aOR	95% CI	aOR	95% CI	aOR	95% CI	aOR	95% CI
Father												
Participated in a home visit from a CHEW where young child feeding was discussed in past 6 mo	16	78	0.8	0.5, 1.3	1.4	0.9, 2.3	1.4	0.8, 2.5	1.4	0.8, 2.5	0.9	0.6, 1.5
Attended a meeting in community where feeding young child feeding was discussed in past 6 mo	13	66	0.7	0.4, 1.1	1.0	0.6, 1.8	1.0	0.5, 1.9	0.8	0.4, 1.3	0.6	0.3, 1.0
Attended a religious service where feeding young child feeding was discussed in past 6 mo	11	56	0.9	0.5, 1.7	1.5	0.8, 2.7	0.9	0.4, 1.9	0.6	0.3, 1.1	0.7	0.4, 1.2
Saw any child feeding information on TV in past 30 d	23	114	1.3	0.8, 2.0	1.1	0.7, 1.7	1.5	0.9, 2.6	0.9	0.5, 1.4	1.2	0.8, 1.9
Heard any child feeding information on radio in past 30 d	26	124	0.8	0.5, 1.3	1.3	0.8, 1.9	1.3	0.8, 2.2	0.9	0.6, 1.5	0.9	0.8, 1.9
Received text messages or voice prompts about child feeding in past 30 d	12	62	1.4	0.8, 2.6	1.5	0.8, 2.5	0.9	0.5, 1.9	1.3	0.7, 2.5	1.3	0.7, 2.1
Mother												
Participated in a home visit from a CHEW where young child feeding was discussed in past 6 mo	21	107	4.0	2.2, 7.1	2.6	1.6, 4.1	3.1	1.9, 5.1	2.1	1.2, 3.6	3.3	2.0, 5.4
Attended a meeting in community where feeding young child feeding was discussed in past 6 mo	12	56	5.1	2.2, 11.9	2.7	1.4, 5.1	2.5	1.3, 4.9	1.3	0.6, 2.6	2.0	1.1, 3.9
Attended a religious service where feeding young child feeding was discussed in past 6 mo	12	56	8.2	3.2, 20.7	2.6	1.4, 4.9	4.8	2.6, 9.0	1.0	0.5, 2.0	2.0	1.1, 3.7
Saw any child feeding information on TV in past 30 d	21	107	2.2	1.3, 3.6	2.1	1.3, 3.3	2.7	1.6, 4.5	1.4	0.8, 2.4	1.7	1.1, 2.7
Heard any child feeding information on radio in past 30 d	16	76	1.2	0.7, 2.0	1.1	0.7, 1.9	0.7	0.4, 1.5	0.7	0.4, 1.3	1.0	0.6, 1.7

1 aOR, adjusted OR; CHEW, community health extension worker; LGA, local government area; TV, television.

2 For fathers, adjusted for age of head of household; household composition counts for adults, children aged <5 y, and school-aged children; sharing a toilet; main source of household's drinking water; type of toilet facility; urbanicity; and fathers' level of education using an inverse probability weighting. For mothers, adjusted for age of head of household; household composition counts for adults, children aged <5 y, and school-aged children; sharing a toilet; main source of household's drinking water; type of toilet facility; urbanicity; and mothers' ethnicity using an inverse probability weighting.

Fathers’ exposure to intervention components was not associated with children's complementary feeding practices (Table 5). Children had higher odds of minimum dietary diversity, consumption of fish, consumption of eggs, and minimum acceptable diet if their mothers reported participating in home visits from a CHEW [minimum dietary diversity: adjusted OR (aOR), 4.0; 95% CI: 2.2, 7.1; fish: aOR, 2.6; 95% CI: 1.6, 4.1; eggs: aOR, 3.1; 95% CI: 1.9, 5.1; minimum acceptable diet: aOR, 3.3; 95% CI: 2.0, 5.4], community meetings (minimum dietary diversity: aOR, 5.1; 95% CI: 2.2, 11.9; fish: aOR, 2.7; 95% CI: 1.4, 5.1; eggs: aOR, 2.5; 95% CI: 1.3, 4.9; minimum acceptable diet: aOR, 2.0; 95% CI: 1.1, 3.9), or religious services where young child feeding was discussed (minimum dietary diversity: aOR, 8.2; 95% CI: 3.2, 20.7; fish: aOR, 2.6; 95% CI: 1.4, 4.9; eggs: aOR, 4.8; 95% CI: 2.6, 9.0; minimum acceptable diet: aOR, 2.0; 95% CI: 1.1, 3.7), or if they saw child feeding information on TV (minimum dietary diversity: aOR, 2.2; 95% CI: 1.3, 3.6; fish: aOR, 2.1; 95% CI: 1.3, 3.3; eggs: aOR, 2.7; 95% CI: 1.6, 4.5; minimum acceptable diet: aOR, 1.7; 95% CI: 1.1, 2.7). Children had higher odds of minimum meal frequency if their mothers reported receiving home visits from a CHEW (aOR: 2.1; 95% CI: 1.2, 3.6).

## Discussion

Although research has shown that fathers are key influencers of IYCF practices, a recent review found that most studies targeting fathers in LMICs have focused on their role in supporting breastfeeding (20). Only a handful of studies included interventions that encouraged fathers to be more involved in their children's complementary feeding and most of these did not quantitatively measure intervention effects (22, 29–32). The present study in Igabi LGA, Kaduna State, Nigeria, engaged fathers in supporting complementary feeding through interpersonal communication, community mobilization, mass media, and mobile phone messaging, and provided information and advice to mothers mainly through interpersonal communication during home visits and mass media. The intervention increased consumption of fish by 8 percentage points, consumption of eggs by 12 percentage points, and minimum meal frequency by 15 percentage points from baseline to endline, but did not increase minimum dietary diversity among children aged 6 to 23 mo. These findings are similar to a study in Kenya where children's animal-source food consumption increased, but minimum dietary diversity did not change in the area where fathers were targeted with the intervention (22). Increasing consumption of fish, eggs, and other animal-source foods in the diets of young children is important because they contain key nutrients that contribute to child growth and development (33, 34). Fish and eggs were among the foods specifically promoted in this study and mothers tended to recall messages related to the promoted foods at endline, which may explain why consumption of these 2 types of animal-source foods increased. However, an increase in the consumption of fish and eggs was not sufficient to modify dietary diversity overall, particularly when consumption of other food groups, such as other fruits and vegetables, decreased.

In this study conducted in Igabi LGA, the prevalence at baseline of minimum dietary diversity (62%) was substantially higher and the prevalence of minimum meal frequency (58%) was substantially lower than measured for Kaduna State in 2017 (22% for minimum dietary diversity, 76% for minimum meal frequency) (23). The baseline sample for the study in Igabi represented the target wards and included approximately 60% participants in urban areas, whereas the baseline sample for the 2017 survey represented the state and included 27% participants in urban areas. Dietary diversity is often higher in urban than rural areas because of better access to markets, higher economic status, and higher parental education (35). This is the case nationally in Nigeria, where the prevalence of minimum dietary diversity is 10% higher in urban than rural areas (27). In the present study, starting from a higher prevalence of minimum dietary diversity may have made it more difficult to achieve an intervention effect, whereas starting from a lower minimum meal frequency may have made it easier to achieve an effect. Another possible explanation for the lack of intervention effects on dietary diversity in this study could be the emphasis on engaging fathers, with fewer intervention components targeted specifically at mothers. Other studies in LMICs, including Alive & Thrive evaluations in other countries, have shown that intervention strategies targeted mainly or entirely at mothers, including counseling during home visits, group nutrition education sessions, cooking demonstrations, and use of print materials and/or mass media, can increase children's dietary diversity or consumption of specific foods (6–13, 36). A study in Kenya showed that targeting fathers in peer-led groups in addition to providing counseling and support to mothers was effective at increasing dietary diversity (22). In settings, like Kaduna, where mothers have primary responsibility for child feeding, it may be necessary to have more intervention touchpoints for mothers, while also engaging fathers, to achieve larger changes in dietary diversity. This theory is supported by the increased odds of minimum dietary diversity among children of mothers who reported intervention exposure in this study.

The percentage of fathers and mothers in this study who reported being exposed to the intervention was low. ICARE estimated that approximately 7250 households in the study area had a father with a child 6–23 mo old at the beginning of the intervention. Based on ICARE's monthly tallies of activities in this population, the percentage of mothers who received a CHEW home visit ranged from 20% to 61% and the percentage of fathers who participated in a CHEW visit ranged from 4% to 13% across the months of the intervention, excluding April to June 2020, when the number of visits was much lower because of coronavirus disease 2019 (COVID-19) restrictions. ICARE's monthly data showed that the percentage of fathers who participated in CBO meetings ranged from 4% to 14% across the months, excluding April and May 2020, when no CBO meetings were held. Participants in sermons by religious leaders were not disaggregated into fathers and mothers of young children, but attendance ranged from 388 to 13,299 monthly participants, excluding April and May 2020, when no sermons were given. ICARE estimated that the percentage of fathers who received mobile phone voice and/or text messages ranged from 53% to 95% across the months. ICARE's monitoring data included a much larger number of households than were surveyed in the evaluation, but the monitoring indicates that the fidelity of the intervention was higher than fathers’ and mothers’ self-reported exposure in the endline surveys indicates. This was most likely related to the pause in several types of intervention activities during the last few months of the intervention period because of COVID-19. Self-reported exposure to Alive & Thrive mass media was the most common type of exposure for fathers and among the most common for mothers. To offset the limited opportunities for face-to-face activities during the pandemic, Alive & Thrive continued supporting regular airing of mass media spots.

Fathers’ self-reported exposure to complementary feeding mobile phone messages was surprisingly low, given that a majority of fathers had mobile phones or access to them and ICARE kept an up-to-date list of eligible fathers to whom they sent the messages. Our endline qualitative data indicated that some fathers were reluctant to read or listen to the messages because they came from an unknown sender. This and other barriers to mHealth participation (37) should be addressed to increase fathers’ exposure to complementary feeding text and voice messages. Very few studies of mHealth approaches in IYCF SBCC have been conducted in LMICs (30, 38), and only 1 included fathers (39). It showed that voice messages to fathers and mothers for 4 wk increased children's consumption of some foods (39). This approach worked over a very short timeframe, but the sustainability of the intervention is unknown. While there are few examples of gamification approaches to mHealth related to nutrition in LMICs (40), mHealth gamification has proven to be effective in high-income countries (41). It could be a more engaging way to educate and involve fathers in child nutrition and should be tested in future studies.

Although the percentage of fathers and mothers exposed to the Alive & Thrive intervention in Igabi LGA was low, we found strong associations between mothers’ specific exposures and complementary feeding practices, but no associations between fathers’ exposures and complementary feeding practices. The intervention encouraged fathers to play a supportive role to mothers in complementary feeding. It is possible that fathers’ intervention exposure influenced child feeding practices indirectly by providing support to mothers to carry out the practices.

Previous research has shown that fathers’ and mothers’ nutrition knowledge contributes to adequate complementary feeding practices (36, 42, 43). In this study, despite low reported exposure to some intervention components, we found substantial improvements between baseline and endline in complementary feeding knowledge among both fathers and mothers. Across multiple food groups, fathers’ and mothers’ knowledge of the timing of introduction of different foods trended downward toward 6 mo, which is the recommended timing (1). In parallel with this knowledge, mothers reported younger ages of actual introduction for many food groups, again pushing the mean ages downward toward 6 mo. Infants need a variety of foods starting from age 6 mo to achieve dietary adequacy, and the late introduction of foods—such as vitamin A–rich fruits and vegetables, animal-source foods, and nuts—deprives them of key nutrients necessary for adequate growth and development (1, 44, 45). Fathers’ and mothers’ knowledge of mean meals per day was above the recommended minimum of 2 times per day for breastfed children aged 6 to 8 mo and 3 times per day for children aged 9 to 23 mo (3).

Nearly all fathers in the study provided support for complementary feeding, as reported by both fathers and mothers. More fathers offered support by providing money for food, and fewer purchased food for the child at endline compared with baseline. Providing money for or purchasing food are the most common and culturally approved roles for fathers in complementary feeding in many African contexts (46–49) and these roles were evident in qualitative data collected for Alive & Thrive in Nigeria in 2017 (24). At endline, more mothers also indicated that fathers offered advice about child feeding, they considered fathers a source of child feeding information or advice, and they said their husband is the person who influences their child feeding practices the most. These findings indicate that more fathers were giving instrumental and informational support to mothers and they align with results from a study to increase fathers’ involvement in complementary feeding in Kenya (22). Both of these studies show that it is feasible to involve some fathers in a variety of roles to support complementary feeding.

### Strengths and limitations

The strengths of this study were the use of formative research to develop key messages and identify acceptable information sources and venues for fathers (i.e., religious leaders, CBO meetings). Another strength was the use of a multipronged approach that included interpersonal communication, community mobilization, and print and mass media. This study also had a few limitations. A cross-sectional pre-post design with no comparison group was selected to align with the available budget. Several differences in the sociodemographic characteristics of participants at baseline and endline were identified and propensity weights were applied in the analysis to account for these differences. It is possible that social desirability bias influenced mothers’ responses to questions about children's dietary intake. However, the lack of change in dietary diversity suggests that social desirability was minimal. Recall bias may have affected fathers’ and mothers’ reports of their intervention exposure, because some activities (especially home visits, CBO meetings, and religious services) had to be paused for a few months before the endline survey due to the COVID-19 pandemic. It is possible that the pandemic affected dietary diversity at endline given its impact on food security globally (50) and a slight increase in food insecurity between baseline and endline was detected.

### Conclusions

This study demonstrates that a multipronged SBCC intervention can modify mothers’ complementary feeding practices, improve fathers’ and mothers’ knowledge of complementary feeding, and increase fathers’ support for complementary feeding, despite low levels of participant-reported exposure to some intervention components. The findings reinforce the importance of involving fathers in programs to improve complementary feeding. They also leave several questions that require further investigation prior to scaling up the intervention in Nigeria, including how to increase intervention exposure and why consumption of some promoted foods (such as fish and eggs) increased, whereas consumption of others (such as vitamin A–rich fruits and vegetables) remained static.

## Supplementary Material

nzac075_Supplemental_File

## Notes

This research was funded by the Alive & Thrive initiative, managed by FHI Solutions, and currently funded by the Bill & Melinda Gates Foundation (OPP 1135932, INV-029432), Irish Aid, the Tanoto Foundation, UNICEF, and the World Bank.

Author disclosures: SK and SB are employed by Alive & Thrive, and AAM is employed by ICARE. They were involved in the design, implementation, and monitoring of the intervention, but not in data collection or analysis for this study. The other authors report no conflicts of interest. The funders had no role in the collection, analysis, or interpretation of the data.

Supplemental Materials 1 and Supplemental Table 1 are available from the “Supplementary data” link in the online posting of the article and from the same link in the online table of contents at https://academic.oup.com/cdn/.

Abbreviations used: aOR, adjusted OR; CBO, community-based organization; CHEW, community health extension worker; COVID-19, coronavirus disease 2019; ICARE, I Care Women and Youth Initiative; IPW, inverse probability weight; IYCF, infant and young child feeding; LGA, local government area; LMIC, low- and middle-income country; SBCC, social and behavior change communication; SMS, short message service; TV, television.
