# Supplementary material for: Complementary Feeding Social and Behavior Change Communication for Fathers and Mothers Improves Children's Consumption of Fish and Eggs and Minimum Meal Frequency in Kaduna State, Nigeria
Source: Curr Dev Nutr. 2022 Apr 8;6(5):nzac075. doi: 10.1093/cdn/nzac075 (PMC9154220; doi:10.1093/cdn/nzac075)
Supplement: nzac075_Supplemental_File [file nzac075_supplemental_file.docx]

**Supplemental Materials 1. Complementary feeding sermon guides, counseling cards, pamphlet, poster, and feeding bowls**


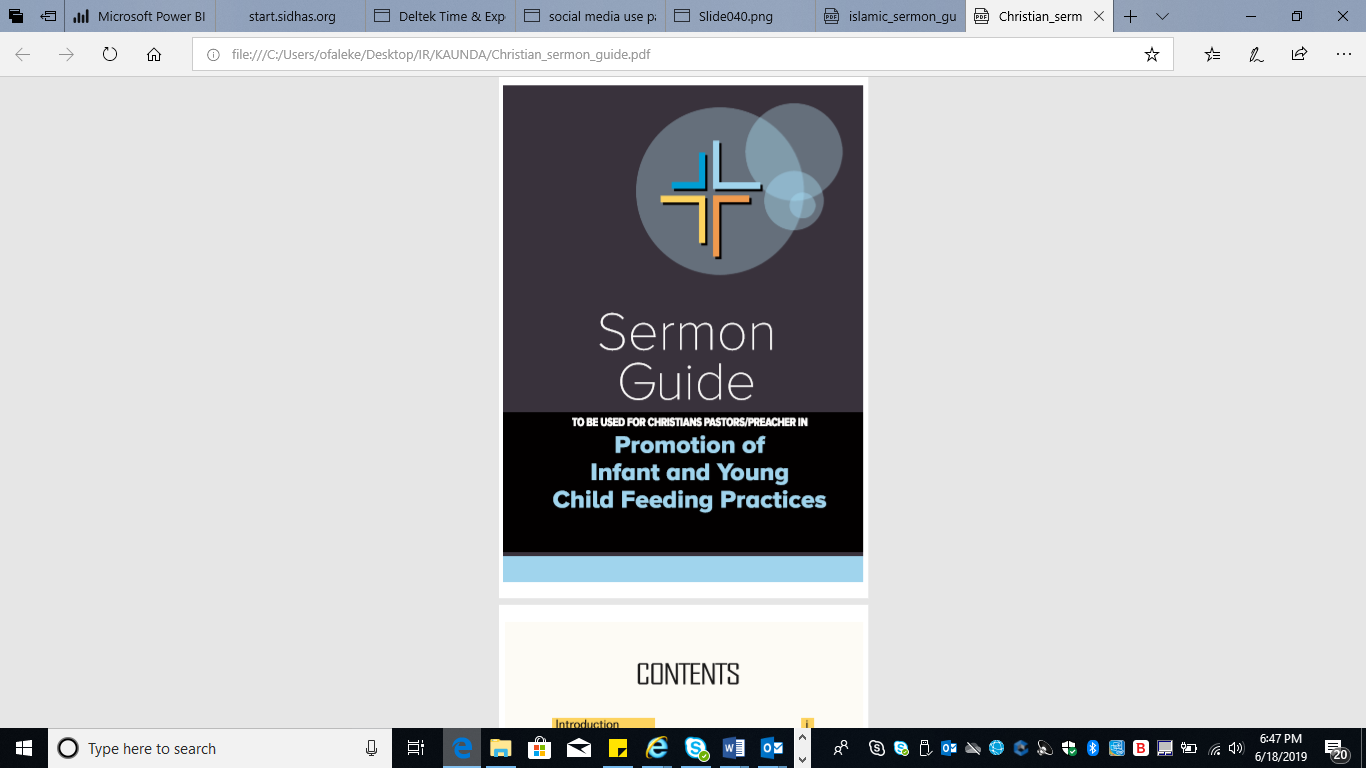

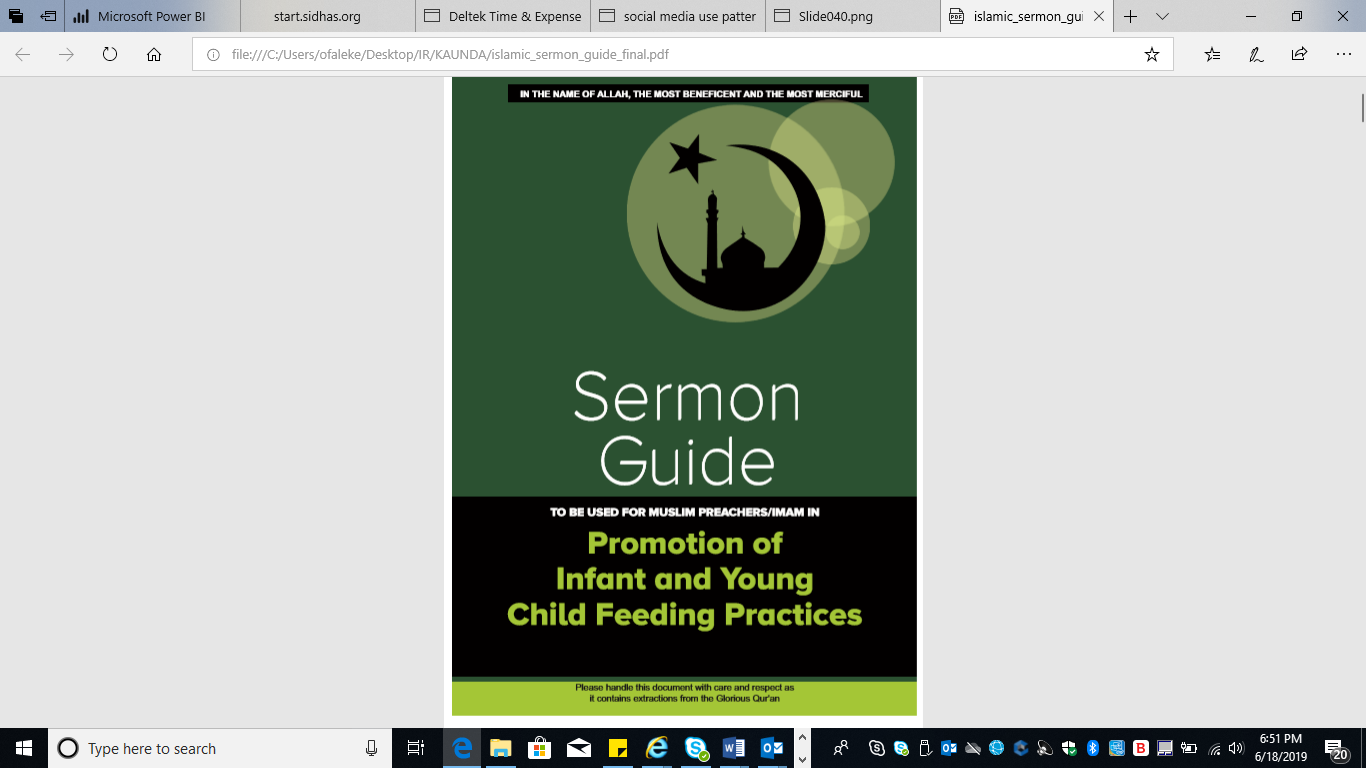


# CHRISTIAN VERSION OF SERMON GUIDE

# Introduction

More than any other civil society representatives, religious leaders who also are spiritual leaders have the experience of establishing and working with diverse social groups and partnerships. Religious leaders are often the most respected figures in their communities. In Nigeria, pastors and priests, play a powerful role in shaping attitudes, opinions, and behaviors because their members trust them. Community members and political leaders listen to religious leaders. More especially religious leaders have the power to raise awareness and influence attitudes, behaviours and practices at the family and community level in line with faith-based teachings. Consequently, it is important that such leaders be well informed about specific dietary diversity practices to be able to motivate their congregations to promote a child’s right to nutritious foods.

Christian religious leaders hold positions of trust. With this trust comes much responsibility—to lead by example, to do the utmost to follow God’s teachings, and to nourish the spiritual and physical well-being of the congregants. Considering this, spiritual leaders have a responsibility to raise awareness within communities about healthy children and child survival and discuss the potential benefits of minimum dietary diversity.

# Sermon Guide

# Dietary Diversity: As God Intended for Healthy Growth

From 6 months onward, breast milk alone is not enough to meet the nutritional requirements of the rapidly growing baby. Consequently, the baby needs other foods in addition to breast milk. Therefore, there is need to introduce other complementary foods.

The body needs different nutrients to function well and different foods provide different nutrients. Eating the right kinds and amounts of food keeps us healthy. No one food supplies all the nutrients the body needs and no one nutrient is most important. Each food group is essential because of the nutrients it provides, and each nutrient depends on other nutrients for it to carry out its proper function. The World Health Organization (WHO) recommends consuming 4 or more designated categories of foods for children under 2 years of age. How then do we make use of these gifts from God?

- Food and Nutrition are divine essential commodities that are very necessary for a solid foundation of life, especially for infants and young children, and for the maintenance of life, sustenance of a healthy level of physical and mental activities, prevention of sickness and enhancing recovery, and sustaining quality of life and producing a quality future generation. But if anyone does not provide for his own, and especially for those of his household, he has denied the faith and is worse than an unbeliever. 1 Timothy 5:8 (KJV)
- As humans, we are among God’s most amazing creations. With our minds and bodies, we can do wonderful things. We may have talents in music, art or storytelling, or we may be skilled craftsmen or farmers. We are all different, and some of us have strengths or weaknesses that others may not have. However, we all need to nourish and protect our bodies. The Apostle Paul says, What? Know ye not that your body is the temple of the Holy Ghost which is in you, which ye have of God, and ye are not your own? For ye are bought with a price: therefore, glorify God in your body, and in your spirit, which are God’s. 1 Corinthians 6:19-20 King James Version (KJV)
- What does Paul mean by this? He is telling us that our body is a temple, which is to be treated with respect and cared for properly. When we take good care of ourselves, we are glorifying God and celebrating His creation. One of the most important ways we can take care of our bodies is to nourish ourselves with nutritious foods.
- One of the most remarkable feats we can accomplish as God’s children is to produce a child. However, this gift comes with responsibility. Fathers, you must take an active role in seeing that the mother and your child are getting the nourishment they need to grow healthy, strong, and sharp. Eating diverse meals, including fruits, vegetables, whole grains and protein, will help keep a mother and growing baby well. God said, See, I have given you every herb that yields seed which is on the face of all the earth, and every tree whose fruit yields seed; to you it shall be for food. Genesis 1:2
- God created us, and He created the food we should use to feed our bodies and treat them like the temples they are intended to be. We have the responsibility to protect our babies from harmful illnesses resulting from malnutrition. As new-born babes, desire the sincere milk of the word, that ye may grow thereby: If so be ye have tasted that the Lord is gracious. Peter 2:2-3 (KJV)

As the trusted spiritual leaders, you should therefore preach to parents:

- Counsel mothers to continue breastfeeding the baby on demand both day and night to meet the nutritional needs of the baby and maintain his/her health and strength.
- Infant from 6 to 9 months:
- Continue feeding the baby with boiled eggs (both white and yolk) or mashed fish

to mashed sweet potatoes or mashed pumpkins and add mashed beans or mashed spinach.

- Feed one-half bowl two times a day.
- Continue breastfeeding.
- Infant from 9 to 11 months:
- Spend time and teach the child to feed herself/himself. Add boiled eggs (both white and yolk) or mashed fish to mashed sweet potatoes or mashed pumpkins and add mashed beans or mashed spinach.
- Feed full bowl 3 times a day.
- Give snacks (such as boiled egg) 1 to 2 times a day.
- Continue breastfeeding.
- Child from 12 to 23 months:
- Encourage child to feed herself/himself. add boiled eggs (both white and yolk) or mashed fish to mashed sweet potatoes or mashed pumpkins and add mashed beans or mashed spinach.
- Feed full bowl 3 times a day.
- Give snacks (such as boiled egg) 1 to 2 times a day.
- Continue breastfeeding.
- Feeding child with poor appetite:
- Feed when the child is hungry.
- Try a variety of foods because child will refuse to eat if given the same food

every time.

- Do not fill the stomach with water, juice, yogurt, etc.
- Encourage and praise child with each mouthful.
- Spend time and be patient while feeding the child.
- Offer child her/his favorite nutritious food.
- Never force feed.
- Continue breastfeeding.
- Feeding a sick child:
- Breastfeed frequently.
- Give favorite nutritious foods.
- Give small, frequent feedings.
- Feed extra amount of nutritious foods for 2 weeks after child recovers until child gains previous weight.

# MUSLIM VERSION OF SERMON GUIDE:

# Introduction

More than any other civil society representatives, religious leaders who are also spiritual leaders have the experience of establishing and working with diverse social groups and partnerships. Religious leaders are often the most respected figures in their communities. In Nigeria, Imams play a powerful role in shaping attitudes, opinions and behaviors because their members trust them. Community members and political leaders listen to religious leaders because they have the power to raise awareness and influence attitudes, behaviors, and practices in line with faith-based teachings. Consequently, it is important that faith-based leaders be well informed about appropriate infant feeding practices to enable them to motivate and counsel their congregations. As the glorious Qur’an stated:

**لَقَدْ خَلَقْنَا الإِنسَانَ فِي أَحْسَنِ تَقْوِيمٍ- (التين:4)**

*We have created man in the best of Moulds*. (Q: 95:4).

Muslim religious leaders hold positions of trust. With this trust comes much responsibility—to lead by example, to do the utmost to follow Allah’s teachings. Imams have the responsibility to raise awareness within communities about healthy children and child survival, and discuss the potential benefits of specific dietary diversity practices. Islam as a complete way of life deals with the concept of infants and young children feeding holistically right from birth to child upbringing. Procreation of the human species is part of the divine plan. Children are therefore entrusted in our hands and we must take care of them in accordance with Islamic injunctions and do all that is humanly possible to protect them from harm, injury, or unnecessary hardship.

The issue of malnutrition knows no religious, tribal, political or any affiliations and the Muslim ummah is not immune to the growing scourge of malnutrition and other preventable diseases that have negative consequences on socioeconomic and political development of the Muslim community and the nation at large. Hence, the need to create awareness on preventing child and maternal malnutrition through Islamic Clerics who are an important influential segment of the Islamic Community. This manual is a guide to assist Imams and preachers to further promote Islamic position on infant and young child feeding practices.

**Sermon Guide**

# Complementary Feeding: For Healthy, Smart, and Strong Growth

From 6 months onward, breast milk alone is not enough to meet the nutritional requirements of the growing baby. Consequently, the baby needs other foods in addition to breast milk. Therefore, there is a need to introduce other complementary foods from 6 months.

The body needs different nutrients to function well and different foods provide different nutrients. Eating the right kinds and amounts of food keeps us healthy. No one food supplies all the nutrients the body needs and no one nutrient is most important. Each food group is essential because of the nutrients that it provides, and each nutrient depends on other nutrients for it to carry out its proper function. Nutrition experts recommend that a child under 2 years of age should eat at least 4 or more designated categories of foods each day to grow healthy and strong.

Islam enjoins Muslim Ummah to eat more vegetables and fruits every day to get the essential vitamins, minerals, and fibers for regulation of body processes. The noble Quran states that:

**وَهُوَ الَّذِي أَنْشَأَ جَنَّاتٍ مَعْرُوشَاتٍ وَغَيْرَ مَعْرُوشَاتٍ وَالنَّخْلَ وَالزَّرْعَ مُخْتَلِفًا أُكُلُهُ وَالزَّيْتُونَ وَالرُّمَّانَ مُتَشَابِهًا وَغَيْرَ مُتَشَابِهٍ كُلُوا مِنْ ثَمَرِهِ إِذَا أَثْمَرَ وَآتُوا حَقَّهُ يَوْمَ حَصَادِهِ وَلَا تُسْرِفُوا إِنَّهُ لَا يُحِبُّ الْمُسْرِفِينَ (سورة الأنعام:141)**

*It is He who produceth Gardens, with trellises and without, and dates, and tilth with produce of all kinds, and olives and pomegranates, similar (in kind) and different (in variety): eat of their fruit in their season but render the dues that are proper on the Day that the harvest is gathered. but waste not by excess: for Allah loveth not the wasters. (Qur’an 6:141)*

**وَهُوَ الَّذِي سَخَّرَ الْبَحْرَ لِتَأْكُلُوا مِنْهُ لَحْمًا طَرِيًّا وَتَسْتَخْرِجُوا مِنْهُ حِلْيَةً تَلْبَسُونَهَا وَتَرَى الْفُلْكَ مَوَاخِرَ فِيهِ وَلِتَبْتَغُوا مِنْ فَضْلِهِ وَلَعَلَّكُمْ تَشْكُرُونَ (14) (سورة النحل:14)**

*It is He who has made the sea subject, that ye may eat thereof flesh that is fresh and tender, and that ye may extract therefrom ornaments to wear; and Thou seest the ships therein that plough the waves, that ye may seek (thus) of the bounty of Allah and that ye may be grateful.*

*(Qur’an 16:14)*

How then do we make use of these gifts from Allah:

- When the child is 6 months old, start introducing foods, such as sweet potatoes, beans, rice, pap, soft porridge, watermelon, mangoes, milk, eggs, carrots and the like, while breastfeeding continues up to 2 years.
- Food and nutrition are divine essential commodities that are very necessary for solid a foundation of life, especially for infants and young children.
- Feeding a child from the four varieties of foods ensures sustenance of a healthy level of physical and mental growth. As stated in the Qur’an:

وَعِنَباً وَقَضْباً (28) وَزَيْتُوناً وَنَخْلاً (29) وَحَدَائِقَ غُلْباً (30) وَفَاكِهَةً وَأَبّاً (31) مَتَاعاً لَكُمْ وَلأَنْعَامِكُمْ (32) (عبس:28-32)

*… and grapes and nutritious plants, and olives and dates, and enclosed Gardens, dense with lofty trees, and Fruits and fodder, for use and convenience to you and your cattle. (Qur’an 80:28-32)*

**وَهُوَ الَّذِي أَنْشَأَ جَنَّاتٍ مَعْرُوشَاتٍ وَغَيْرَ مَعْرُوشَاتٍ وَالنَّخْلَ وَالزَّرْعَ مُخْتَلِفًا أُكُلُهُ وَالزَّيْتُونَ وَالرُّمَّانَ مُتَشَابِهًا وَغَيْرَ مُتَشَابِهٍ كُلُوا مِنْ ثَمَرِهِ إِذَا أَثْمَرَ وَآتُوا حَقَّهُ يَوْمَ حَصَادِهِ وَلَا تُسْرِفُوا إِنَّهُ لَا يُحِبُّ الْمُسْرِفِينَ (سورة الأنعام:141)**

*It is He who produceth Gardens, with trellises and without, and dates, and tilth with produce of all kinds, and olives and pomegranates, similar (in kind) and different (in variety): eat of their fruit In their season, but render the dues that are proper on the Day that the harvest is gathered. but waste not by excess: for Allah loveth not the wasters (Qur’an 6:141)*

**وَهُوَ الَّذِي سَخَّرَ الْبَحْرَ لِتَأْكُلُوا مِنْهُ لَحْمًا طَرِيًّا وَتَسْتَخْرِجُوا مِنْهُ حِلْيَةً تَلْبَسُونَهَا وَتَرَى الْفُلْكَ مَوَاخِرَ فِيهِ وَلِتَبْتَغُوا مِنْ فَضْلِهِ وَلَعَلَّكُمْ تَشْكُرُونَ (14) (سورة النحل:14)**

*It is He who has made the sea subject, that ye may eat thereof flesh that is fresh and tender, and that ye may extract therefrom ornaments to wear; and Thou seest the ships therein that plough the waves, that ye may seek (thus) of the bounty of Allah and that ye may be grateful. (Qur’an 16:1)4*

**Key Khutbah Message**

As the trusted Spiritual Leaders, you should therefore:

- Urge fathers and mandate them to take responsibility for their family and provide the variety of food that the child needs to grow healthy. Allah SWT, said:

وَعَلَى الْمَوْلُودِ لَهُ رِزْقُهُنَّ وَكِسْوَتُهُنَّ بِالْمَعْرُوفِ (البقرة:233)

*… but He shall bear the cost of their food and clothing on equitable terms … .* (Quran 2:233).

- Advise mothers that from 6 to 9 months, babies should be fed soft, mashed foods at least 2 times per day. Food should be thick, not watery; and to continue breastfeeding the baby on demand both day and night to meet the nutritional needs of the baby.

In conclusion, The Prophet Muhammad (SAW) says:

"من رأى منكم منكرا فليغيره بيده، فإن لم يستطع فبلسانه، فإن لم يستطع فبقلبه وذلك أضع أضعف الإيمان (صحيح مسلم: ،

*If one of you sees something wrong, let him change it with his hand (Strength, resources, etc.), if he cannot, then with his tongue (speaking up against it), if he cannot, then with his heart (condemning in his heart and not partaking in it) and this is the weakest faith.*

– Prophet Muhammad (S.A.W.) narrated by Abu said, reported by Muslim.

As the trusted spiritual leaders, you should therefore preach to parents:

- Counsel mothers to continue breastfeeding the baby on demand both day and night to meet the nutritional needs of the baby and maintain his/her health and strength.
- Infant from 6 to 9 months:
- Continue feeding the baby with boiled eggs (both white and yolk) or mashed fish to mashed sweet potatoes or mashed pumpkins and add mashed beans or mashed spinach.
- Feed one-half bowl 2 times a day.
- Continue breastfeeding.
- Infant from 9 to 11 months:
- Spend time and teach a child to feed herself/himself. Add boiled eggs (both white and yolk) or mashed fish to mashed sweet potatoes or mashed pumpkins and add mashed beans or mashed spinach.
- Feed full bowl 3 times a day.
- Give snacks (such as boiled egg)1 to 2 times a day.
- Continue breastfeeding.
- Child from 12 to 23 months:
- Encourage a child to feed herself/himself. Add boiled eggs (both white and yolk) or mashed fish to mashed sweet potatoes or mashed pumpkins and add mashed beans or mashed spinach
- Feed full bowl 3 times a day.
- Give snacks (such as boiled egg)1 to 2 times a day.
- Continue breastfeeding.
- Feeding child with poor appetite:
- Feed when the child is hungry.
- Try a variety of foods because child will refuse to eat if given the same food

every time.

- Do not fill the stomach with water, juice, yogurt, etc.
- Encourage and praise child with each mouthful.
- Spend time and be patient while feeding the child.
- Offer child her/his favorite nutritious food.
- Never force feed.
- Continue breastfeeding.
- Feeding a sick child:
- Breastfeed frequently
- Give favorite nutritious foods.
- Give small, frequent feedings.
- Feed extra amount of nutritious foods for 2 weeks after child recovers until child gains previous weight

**Counseling Cards**


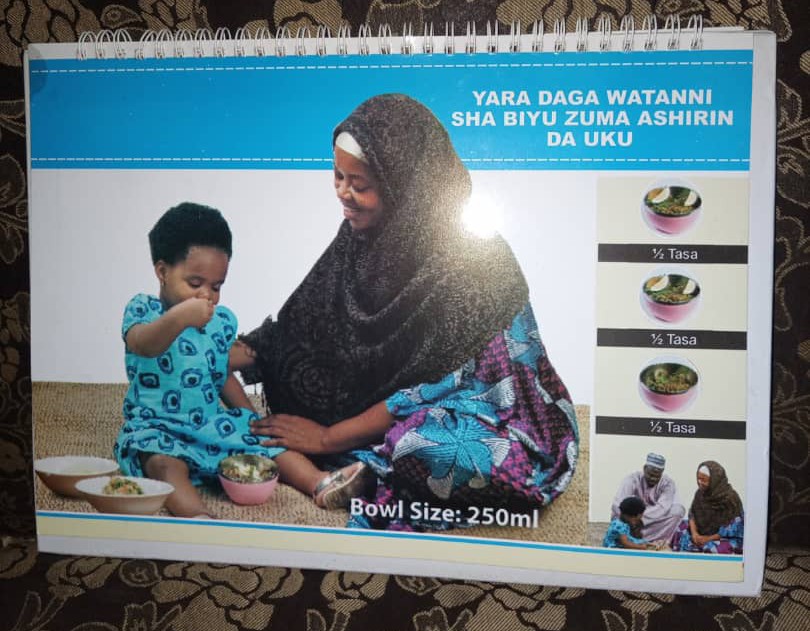

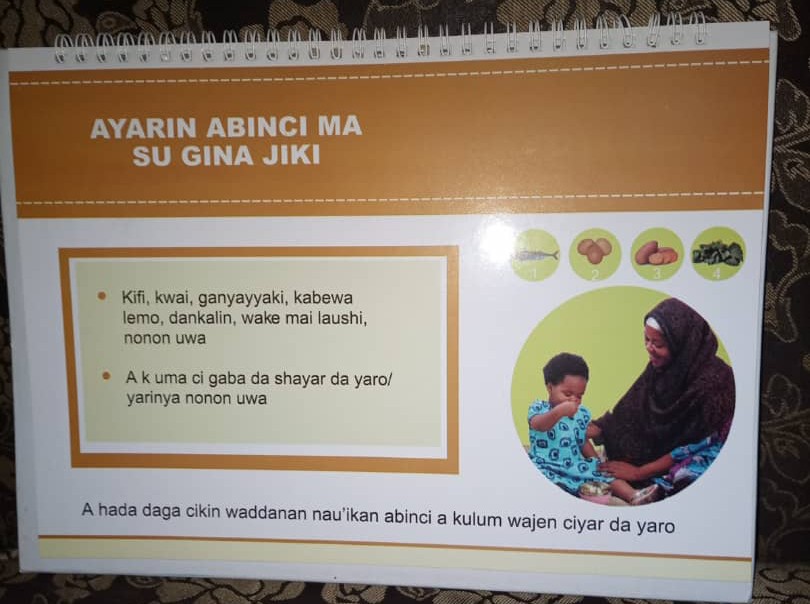


**Pamphlet**


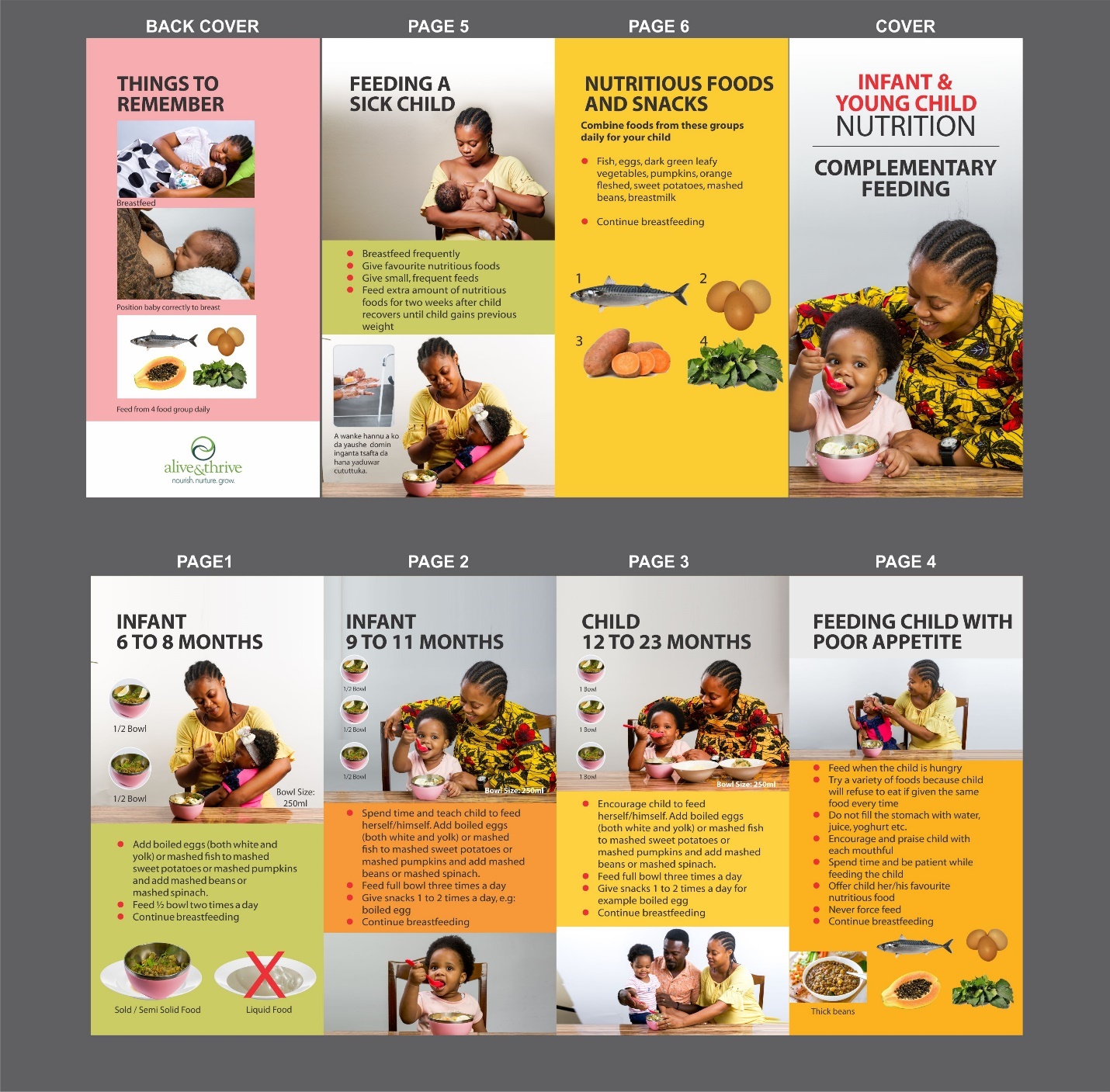


**Poster**


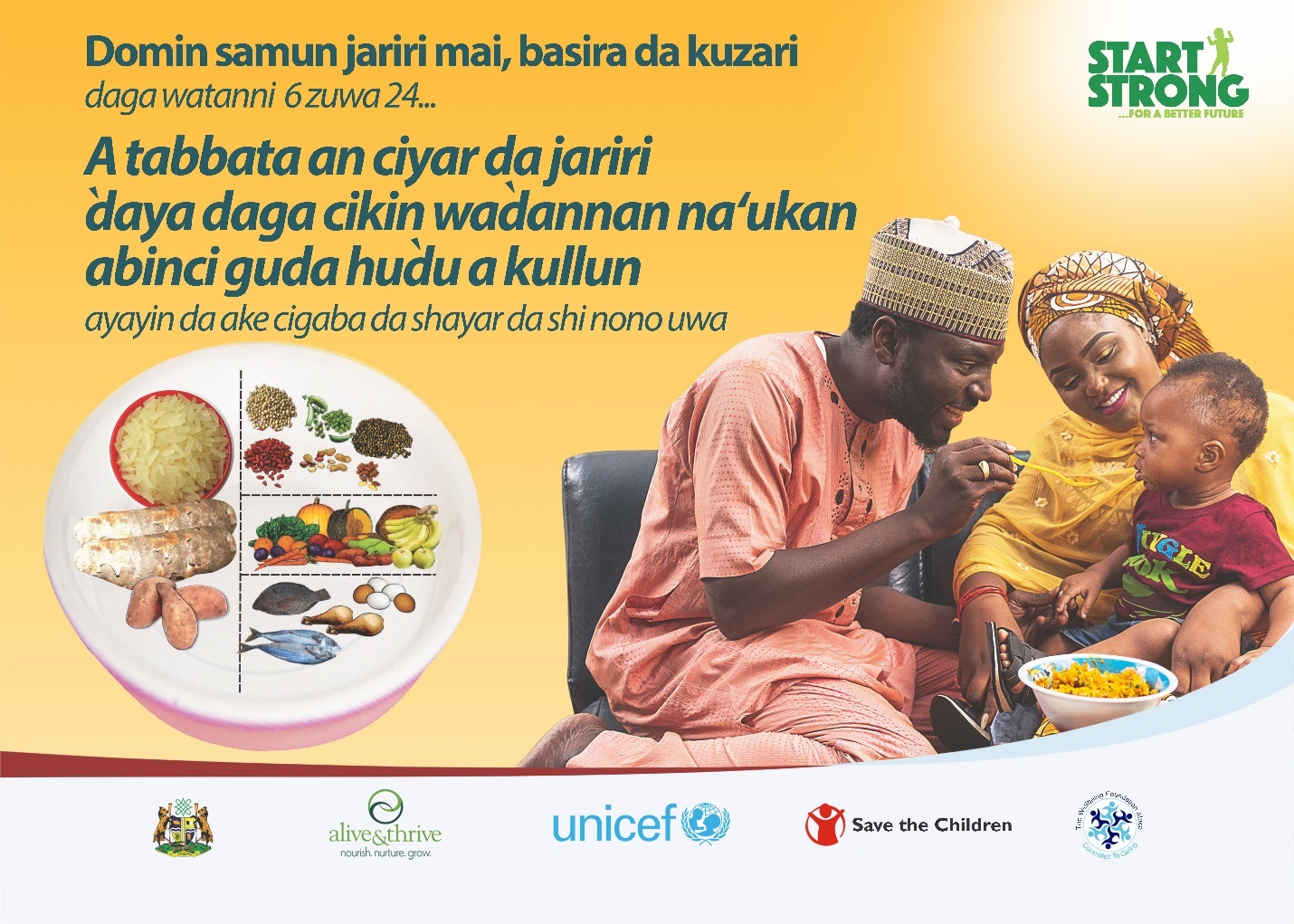


**Feeding Bowls**


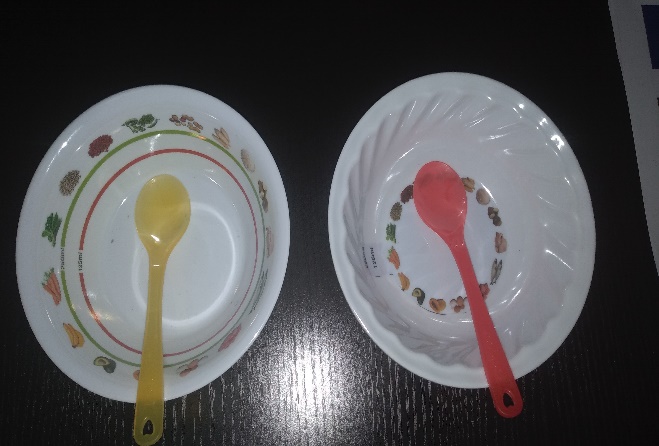


**Supplemental Table 1. Minimum Dietary Diversity SMS Messages and Voice Prompts**

| **SMS Messages** |
| --- |
| ENGLISH LANGUAGE MDD SMS   - From 6 months, provide foods such as fish, egg, pumpkin, beans, sweet potatoes, fruits and vegetables. Enough time should be given to feeding your child daily.   HAUSA LANGUAGE MDD SMS   - Daga watanni shida a samama yaro abinci daga cikin kifi, kwai, kabewa, wake, dankalin hausa, y’ay’an itatuwa da gangaryaki. Uwa ta sami lokaci wajen ciyar da yaro   PIDGIN LANGUAGE MDD SMS   - Buy fish, egg, pumpkin, beans, sweet potato, fruit and vegetable dey give am to your pikin wey reach 6 months. Make mama get plenty time to give am this beta food. |
| **Voice Prompts** |
| ENGLISH LANGUAGE MDD VOICE PROMPT   - Dear father, for healthy growth of your child, from 6 months, provide additional nutritious foods such as fish, egg, pumpkin, beans, sweet potatoes, fruits, and vegetables for the baby. - Also ensure the mother continues breastfeeding the baby in addition to frequently feeding the child with nutritious foods every day. This message is from the Kaduna State Government with support from Alive & Thrive and the I Care Women and Youth Initiative.   HAUSA LANGUAGE MDD VOICE PROMPT   - Gaisuwa da fatan alheri. Domin inganta koshin lafiyar yaro, daga wata shida da haihuwa, A samar masa abinci mai gina jiki da ya hada da kamar su kifi, kwai, kabewa, wake, dankalin Hausa, da y’sy’an itatuwa, da gangaryaki. - Tabbattar an ci gaba da shayar da yaron nonon uwa tare da cigaba da ciyarda shi nau’uksn abinci a kai-akai a kulun. Wannan sakon da Gwamnatin Kaduna tare da tallafin Alive and Thrive da kuma I Care Women and Youth Initiative.   PIDGIN LANGUAGE MDD VOICE PROMPT MESSAGES   - Papa wey like him pikin, for ur pikin to grow beta and dey kakaraka pass ur neighbour pikin oo, buy am fish, egg, pumpkin, beans, sweet potato, fruit and vegetable and dey give am to your pikin wey don pass 6 months wey go helep am make e grow well well ooo... - As Papa wey care, make sure say dem take plenty time to give am all dis beta food dem...And make una always dey give am food anytime hunger wire am oooh !!!. This na from the Kaduna State Government with join body from Alive & Thrive and I Care Women and Youth Initiative. |
